# Supplementary material for: Genes Implicated in Familial Parkinson’s Disease Provide a Dual Picture of Nigral Dopaminergic Neurodegeneration with Mitochondria Taking Center Stage
Source: Int J Mol Sci. 2021 Apr 28;22(9):4643. doi: 10.3390/ijms22094643 (PMC8124903; doi:10.3390/ijms22094643)
Supplement: Supplementary file 1 [file ijms-22-04643-s001.zip › ijms-1182801-supplementary.pdf]

Supplementary Material: The following are available online at www.mdpi.com/xxx/s1,

Genes implicated in familial Parkinson's disease provide a dual picture of nigral dopaminergic neurodegeneration, with mitochondria taking center stage

Rafael Franco‡[1,2], Rafael Rivas-Santisteban‡[1,2], Gemma Navarro‡[2,3], Annalisa Pinna*‡[4] and Irene Reyes-Resina‡[1†]

1 Dept. Biochemistry and Molecular Biomedicine. University of Barcelona, 08028 Barcelona, Spain; rfranco@ub.edu (RF); rrivasbioq@gmail.com (RR-S); ire-reyes@hotmail.com (IR-R)

2 Centro de investigación Biomédica en Red Enfermedades Neurodegenerativas (CiberNed), Instituto de Salud Carlos III, 28031 Madrid, Spain

3 Dept. Biochemistry and Physiology. School of Pharmacy and Food Sciences. University of Barcelona, 08028 Barcelona, Spain; g.navarro@ub.edu (GN)

4 National Research Council of Italy (CNR), Neuroscience Institute – Cagliari; Cittadella Universitaria, 09042 Monserrato (CA), Italy. apinna@unica.it (AP)

† Current address: RG Neuroplasticity, Leibniz Institute for Neurobiology, Brenneckestr. 6, 39118 Magdeburg, Germany

‡ These authors contributed equally to this work.

*** Corresponding author**

Annalisa Pinna: National Research Council of Italy (CNR), Neuroscience Institute – Cagliari; Cittadella Universitaria, Blocco A, SP 8, Km 0.700, 09042 Monserrato (Cagliari), Italy. apinna@unica.it (AP); annalisa.pinna@in.cnr.it (AP)

Supplementary Figure S1 and Table S1

Supplementary Figure S1.
STRING analysis of connections of either SNCA (A), GBA (B), FBXO7 (C), ATP13A2 (D) and UCHL1 (E).
Line color code. Sky blue: known interactions from curated databases; magenta: experimentally determined interactions; green: predicted from neighborhood; red: predicted from gene fusions; blue: predicted from gene co-occurrence; pastel green: textmining; black: coexpression and clear violet: protein homology.

Panel A: SNCA

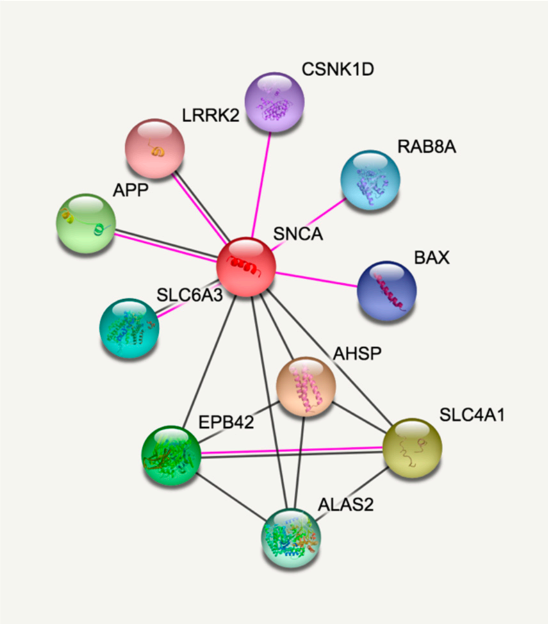

Panel C: FBXO7

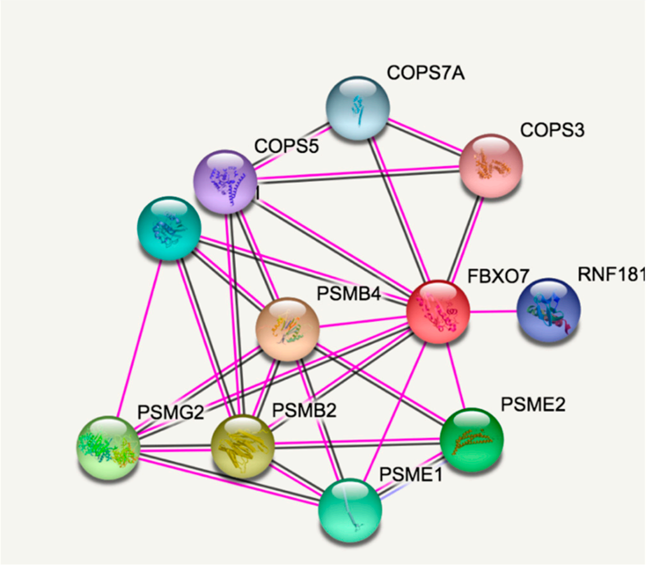

Panel B: GBA

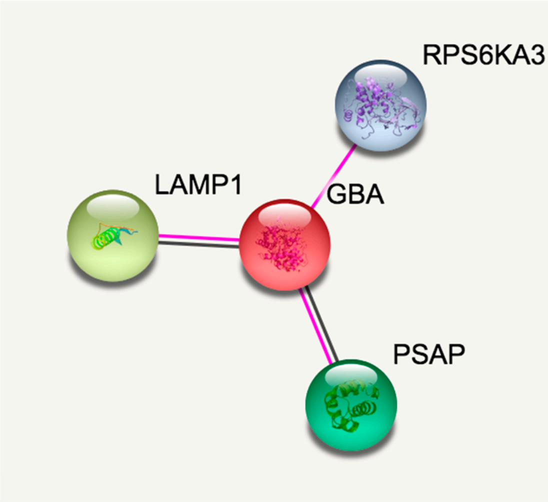

Panel D: ATP13A2

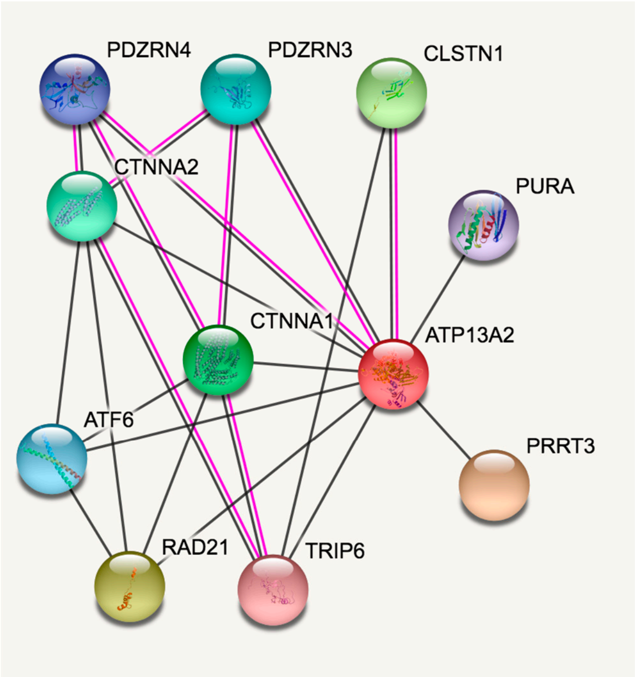

Panel E: UCHL1

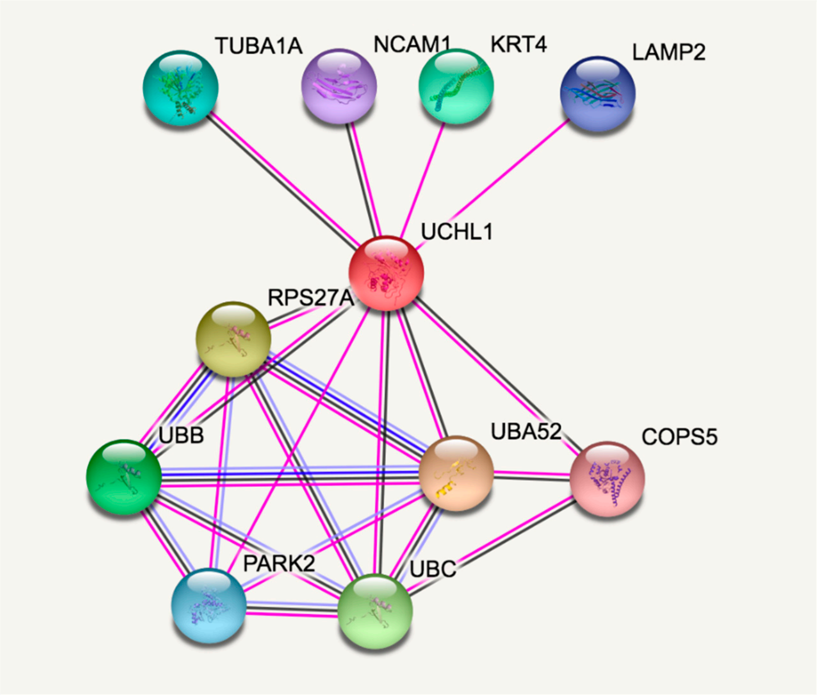

Table S1. Findings in animal models related to Gba, Uchl1, *Vps35, Atp13a2, Pla2g6, Dnajc6, Synj1, DJ-1/Park7* and *Fbxo7*

Gene	Animal model(s)	*In vivo*	*In vitro*	Expression level	Main findings	Reference
GBA	K-14Cre-positive Gba$^{lnl/lnl}$		X	*Gba* KO mice (except in skin)	• Strong similarities with Gaucher disease pathology	[1]
	B6;129S6-*Gba*^{tm1Nsb}/J		X	*Gba* KO mice	• Downregulation of neurotrophic factors	[2]
	n.s.n.		X	*Gba* KO mice	• Autophagic lysosome reformation dysfunction	[3]
	Mice resulting from crossing *SNCA* Tg/Tg line with *Gba* +/- line (n.s.n.)		X	*SNCA* overexpressing *Gba* KO mice	• Increased total α -syn accumulation<br>• Altered lipid metabolism	[4]
	Mice resulting from crossing A53T α-synuclein transgenic line M83 with *Gba* KO line B6;129S6-*Gba*^{tm1Nsb}/J	X	X	A53T-*SNCA* overexpressing *Gba* KO mice	• Impact on PD disease onset	[5]
	B6;129S4-Gbatm1Rlp/Mmnc	X	X	L444P-*Gba* KI mice	• Increased total α -syn accumulation<br>• Enhanced neuron vulnerability	[6]
	B6;129P2-*Uchl1*^{tm1Dgen}/Mmnc	X	X	*UCH-L1* KO mice	• Significantly decreased motor performance on the rotarod test	[7]
UCHL1>	Mice resulting from crossing B6;129P2-*Uchl1*^{tm1Dgen}/Mmnc line with Thy1-maSN line	X	X	*Snca* overexpressing *Uch-L1* KO mice	• Earlier-onset motor deficits	[8]
	B6.Cg-*Vps35*$^{tm1.2Mjff}$/J	X	X	D620N-*Vps35* KI mice	• Manifest tau neuropathology and dopaminergic neurodegeneration	[9]

	129S-*Prkn*^{tm1Rpa}/J	X		*Prkn* KO mice	• Insensibility to 6-OHDA and methamphetamine neurotoxicity	[10]
VPS35	129S-*Prkn*^{tm1Rpa}/J	X		*Prkn* KO mice	• Do not exhibit robust signs of parkinsonism	[11]
ATP13A2	Mice resulting from crossing B6N.129S6(Cg)-*Atp13a2*^{tm1Pjsch}/J line with B6;DBA-Tg(Thy1-SNCA)61Ema line	X		*Atp13a2* KO mice overexpressing human *SNCA*	• Impaired sensorimotor function	[12]
	B6;129S6-*Pla2g6*^{tm1Zyao}/J	X	X	*Pla2g6* KO mice	• No evidence that leads to dyslipidemia<br>• Altered catabolism of TAG-rich lipoproteins	[13]
	iPLA2-VIA–deficient flies (n.s.n.)	X	X	*iPLA2-VIA* KO Drosophila	• Dysregulation in neuronal functions and α-syn<br>1. stability	[14]
	y w;; iPLA2-VIAΔ174<br>y w;; iPLA2-VIAΔ192	X	X	*iPLA2-VIA* KO Drosophila	2. Impaired retromer and lysosomal function in neurons	[15]
PLA2G6	PLA2G6$^{D331Y/D331Y}$	X	X	D331Y-*Pla2g6* KI mice	• Mitophagy impairment<br>• Mitochondrial dysfunction<br>3. ER stress	[16]
	B6.129-*Dnajc6*^{tm1Legr}/Mmjax		X	*Dnajc6* KO mice	• Endocytosis and clathrin-uncoating defects<br>4. at synapses	[17]
	C57BL/6-*Dnajc6*^{em1Mcook}/J	X	X	R857G-*Dnajc6* KI mice	• Impaired clathrin-mediated<br>5. trafficking at the Golgi and at the synapse	[18]
	UAS-aux^{R16182} (aux RNAi, VDRC)<br>UAS-aux^{R103426} (aux RNAi, VDRC)	X	X	Reduced aux expression Drosophila	• Reduced locomotion and longevity<br>• DA neuron loss at the PPM1/2 cluster<br>• Enhanced and accelerated α-syn-mediated DA<br>6. neuron loss	[19]
DNAJC6	B6;129-*Synj1*^{tm1Pdc}/J		X	*Synj1* KO mice	7. Synaptic vesicles still form from synaptic endosomes	[20]
	B6;129-*Synj1*^{tm1Pdc}/J		X	*Synj1* KO mice	8. Defects in both endocytosis and post-endocytic vesicle	[21]
	B6;129-*Synj1*^{tm1Pdc}/J		X	*Synj1* KO mice	• Exhibit neurological defects<br>9. Die shortly after birth.	[22]
SYNJ1	B6;129-*Synj1*^{tm1Pdc}/J		X	*Synj1* KO mice	10. Larger excitatory postsynaptic amplitudes	[23]
	Mice resulting from crossing B6;129-*Synj1*^{tm1Pdc}/J line with AD model line Tg2576	X	X	*Synj1* KO + AD model mice	11. Ameliorated AD-associated behavioral and synaptic deficits	[24]
	Mice resulting from crossing B6;129-*Synj1*^{tm1Pdc}/J line and B6.Cg-Tg(Lrrk2*G2019S)2Yue/J line	X	X	G2019S-*Lrrk2* overexpressing *Synj1* KO mice	• Impaired sustained exocytosis in MB neurons<br>12. Altered specific motor functions	[25]
	n.s.n.		X	Overexpressing *Synj1* mice	13. Enlarged endosomes	[26]
	n.s.n.	X	X	Down ‘s syndrome mouse model overexpressing either *Synj1* or human *SYNJ1*	• Altered behavior<br>14. Brain dysfunction	[27]
	SJ1 ^{RQ}-KI	X	X	R259Q-*Synj1* KI mice	• Endocytic defects<br>15. Elevated auxilin and parkin	[28]
	B6.129-*Park7*^{tm1Mak}	X	X	*Dj-1* KO mice	16. Impaired running wheel and rotarod performance	[29]
	B6.129P2-*Park7*^{tm1Dsp}/Cnbc	X	X	*Dj-1* KO mice	• Mitochondrial defects and more ROS production<br>17. Altered autophagy	[30]
	B6.129P2-*Park7*^{tm1Dsp}/Cnbc	X	X	*Dj-1* KO mice	18. Increased sensitivity to excitotoxicity and ischemia	[31]
	B6.129P2-*Park7*^{tm1Dsp}/Cnbc	X	X	*Dj-1* KO mice	• DJ-1 is crucial for full	[32]

					19. activation of AKT upon oxidative injury	
	B6.Cg-*Park7*^{tm1Shn}/J		X	*Dj-1* KO mice	20. Lower expression of UCP4	[33]
	B6.Cg-*Park7*^{tm1Shn}/J		X	*Dj-1* KO mice	21. Loss of SNc dopaminergic neurons.	[34]
	B6.Cg-*Park7*^{tm1Shn}/J	X	X	*Dj-1* KO mice	22. Mitochondrial oxidative stress	[35]
	B6.Cg-*Park7*^{tm1Shn}/J		X	*Dj-1* KO mice	• Increased mitochondrial Trx activity<br>• Increased GSH and GSSG levels<br>• Increased mitochondrial glutaredoxin (GRX)<br>23. activity	[36]
	B6.Cg-*Park7*^{tm1Shn}/J		X	*Dj-1* KO mice	• Declined dendritic complexity<br>24. Loss of dendritic spines in striatal MSNs	[37]
	B6.Cg-*Park7*^{tm1Shn}/J		X	*Dj-1* KO mice	• Mitochondrial respiration not affected<br>25. Increased ROS production	[38]
	B6.Cg-*Park7*^{tm1Shn}/J		X	*Dj-1* KO mice	• Altered Ca^{2+} homeostasis in the skeletal muscle<br>26.	[39]
	B6.Cg-*Park7*^{tm1Shn}/J	X	X	*Dj-1* KO mice	• Absence of dopaminergic neuronal degeneration<br>27. Oxidative damage	[40]
DJ-1/PARK7	B6.Cg-*Park7*^{tm1Shn}/J	X	X	*Dj-1* KO mice	• Absence of LTD in medium spiny neurons<br>28. Hypoactivity in the open field	[41,42]
	B6.Cg-*Park7*^{tm1Shn}/J	X	X	*DJ-1* KO mice	• Enhanced sensitivity to energy metabolism<br>29. impairment	[43]
	B6;129X1-*Park7*^{tm1Cai}/Mmjax	X	X	*Dj-1* KO mice	• Retinal abnormalities<br>• Visual dysfunction<br>30. Increased oxidative stress in mice	[44]
	B6;129X1-*Park7*^{tm1Cai}/Mmjax	X	X	*Dj-1* KO mice	31. Progressive behavioral deficits	[45]
	DAT-Ret;DJ-1 mice resulting from crossing B6.Cg-*Park7*^{tm1Shn}/J line with DAT-Ret line	X	X	*Dj-1* KO + midbrain dopaminergic neurons *Ret* KO mice	• Interaction between DJ-1 and Ret-mediated signaling<br>• DJ-1 promotes cell<br>32. survival	[46]
	B6.129-*Park7*^{tm1Mak}<br>FVB/N-*Khdrbs2*$^{Tg(LRRK2*R1441G)135Cjli}$/J	X	X	-*Dj-1* KO mice<br>-R1441G-*LRRK2* expressing mice	• Modest impairments of motor behavior<br>33. No dysfunctions of DA overflow or reuptake	[47]
	B6.Cg-*Park7*^{tm1Shn}/J		X	-*Dj-1* KO mice<br>-*DJ-1*-deficient SH-SY5Y cells	• Inhibited S-nitrosylation of endogenous Parkin<br>• Mitochondrial<br>34. dysfunction	[48]
	B6.Cg-*Park7*^{tm1Shn}/J<br>B6.129S4-*Park2*^{tm1Shn}/J		X	-*Dj-1* KO mice<br>-*Prkn* KO mice	• Changes in the expression of neurotransmitter<br>35. receptors	[49]
	Mice resulting from crossing B6.129S4-*Prkn*^{tm1Shn}/J line with B6.Cg-*Park7*^{tm1Shn}/J line and *Sod1* KO or *Sod2* KO line	X	X	Triple *Prkn* KO + *Dj-1* KO + *Sod1* or *Sod2* KO mice	• Enhanced performance in locomotor tests<br>36. Elevated levels of dopamine in the striatum	[50]
	n.s.n.	X	X	*DJ-1* β mutants Drosophila	37. Oxidative stress-sensitive locomotive dysfunction	[51]
FBXO7	n.s.n.	X	X	*Fbxo7* KO mice	• Reduced proteasome activity<br>• Motor deficits<br>• Premature death<br>38. Vital role for FBXO7 in neurons	[52]

n.s.n.: Non standard nomenclature

References in table S1:

1. Enquist, I. B.; Bianco, C. Lo; Ooka, A.; Nilsson, E.; Månsson, J. E.; Ehinger, M.; Richter, J.; Brady, R. O.; Kirik, D.; Karlsson, S. Murine models of acute neuronopathic Gaucher disease. *Proc. Natl. Acad. Sci. U. S. A.* **2007**, *104*, 17483–17488.
2. Eun, Y. K.; Young, B. H.; Sang, H. G.; Lee, B.; Jung, S. C. Downregulation of neurotrophic factors in the brain of a mouse model of Gaucher disease; implications for neuronal loss in Gaucher disease. *Exp. Mol. Med.* **2006**, *38*, 348–356.
3. Magalhaes, J.; Gegg, M. E.; Migdalska-Richards, A.; Doherty, M. K.; Whitfield, P. D.; Schapira, A. H. V. Autophagic lysosome reformation dysfunction in glucocerebrosidase deficient cells: Relevance to Parkinson disease. *Hum. Mol. Genet.* **2016**, *25*, 3432–3445.
4. Ikuno, M.; Yamakado, H.; Akiyama, H.; Parajuli, L. K.; Taguchi, K.; Hara, J.; Uemura, N.; Hatanaka, Y.; Higaki, K.; Ohno, K.; Tanaka, M.; Koike, M.; Hirabayashi, Y.; Takahashi, R. GBA haploinsufficiency accelerates α-synuclein pathology with altered lipid metabolism in a prodromal model of Parkinson's disease. *Hum. Mol. Genet.* **2019**, *28*, 1894–1904.
5. Tayebi, N.; Parisiadou, L.; Berhe, B.; Gonzalez, A. N.; Serra-Vinardell, J.; Tamargo, R. J.; Maniwang, E.; Sorrentino, Z.; Fujiwara, H.; Grey, R. J.; Hassan, S.; Blech-Hermoni, Y. N.; Chen, C.; McGlinchey, R.; Makariou-Pikis, C.; Brooks, M.; Ginns, E. I.; Ory, D. S.; Giasson, B. I.; Sidransky, E. Glucocerebrosidase haploinsufficiency in A53T α-synuclein mice impacts disease onset and course. *Mol. Genet. Metab.* **2017**, *122*, 198–208.
6. Migdalska-Richards, A.; Wegrzynowicz, M.; Rusconi, R.; Deangeli, G.; Di Monte, D. A.; Spillantini, M. G.; Schapira, A. H. V. The L444P Gba1 mutation enhances α-synuclein induced loss of nigral dopaminergic neurons in mice. *Brain* **2017**, *140*, 2706–2721.
7. Chen, F.; Sugiura, Y.; Myers, K. G.; Liu, Y.; Lin, W. Ubiquitin carboxyl-terminal hydrolase L1 is required for maintaining the structure and function of the neuromuscular junction. *Proc. Natl. Acad. Sci. U. S. A.* **2010**, *107*, 1636–1641.
8. Shimshek, D. R.; Schweizer, T.; Schmid, P.; Van Der Putten, P. H. Excess α-synuclein worsens disease in mice lacking ubiquitin carboxy-terminal hydrolase L1. *Sci. Rep.* **2012**, *2*.
9. Chen, X.; Kordich, J. K.; Williams, E. T.; Levine, N.; Cole-Strauss, A.; Marshall, L.; Labrie, V.; Ma, J.; Lipton, J. W.; Moore, D. J. Parkinson's disease-linked D620N VPS35 knockin mice manifest tau neuropathology and dopaminergic neurodegeneration. *Proc. Natl. Acad. Sci. U. S. A.* **2019**, *116*, 5765–5774.
10. Perez, F. A.; Curtis, W. R.; Palmiter, R. D. Parkin-deficient mice are not more sensitive to 6-hydroxydopamine or methamphetamine neurotoxicity. *BMC Neurosci.* **2005**, *6*, 1–10.
11. Perez, F. A.; Palmiter, R. D. Parkin-deficient mice are not a robust model of parkinsonism. *Proc. Natl. Acad. Sci. U. S. A.* **2005**, *102*, 2174–2179.
12. Dirr, E. R.; Ekhator, O. R.; Blackwood, R.; Holden, J. G.; Masliah, E.; Schultheis, P. J.; Fleming, S. M. Exacerbation of sensorimotor dysfunction in mice deficient in Atp13a2 and overexpressing human wildtype α-synuclein. *Behav. Brain Res.* **2018**, *343*, 41–49.
13. Zhang, L.; Zhong, S.; Li, Y.; Ji, G.; Sundaram, M.; Yao, Z. Global Inactivation of the Pla2g6 Gene in Mice Does Not Cause Dyslipidemia under Chow or High-fat Diet Conditions. **2013**, *18*.
14. Mori, A.; Hatano, T.; Inoshita, T.; Shiba-fukushima, K.; Koinuma, T.; Meng, H. regulates neuronal functions and α -synuclein stability through membrane remodeling. **2019**, 1–11.
15. Lin, G.; Lee, P. T.; Chen, K.; Mao, D.; Tan, K. L.; Zuo, Z.; Lin, W. W.; Wang, L.; Bellen, H. J. Phospholipase PLA2G6, a Parkinsonism-Associated Gene, Affects Vps26 and Vps35, Retromer Function, and Ceramide Levels, Similar to α-Synuclein Gain. *Cell Metab.* **2018**, *28*, 605-618.e6.
16. Chiu, C.; Lu, C.; Weng, Y.; Chen, Y.; Huang, Y. PARK14 (D331Y) PLA2G6 Causes Early-Onset Degeneration of Substantia Nigra Dopaminergic Neurons by Inducing Mitochondrial Dysfunction , ER Stress , Mitophagy Impairment and Transcriptional Dysregulation in a Knockin Mouse Model. **2018**, *14*.

17. Yim, Y.; Sun, T.; Wu, L.; Raimondi, A.; Camilli, P. De; Eisenberg, E.; Greene, L. E. Endocytosis and clathrin-uncoating defects at synapses of auxilin knockout mice. **2010**, *107*, 1–6.
18. Roosen, D. A.; Landeck, N.; Conti, M.; Smith, N.; Saez-atienzar, S. Mutations in Auxilin cause parkinsonism via impaired clathrin-mediated trafficking at the Golgi apparatus and synapse. **2019**, 1–39.
19. Song, L.; He, Y.; Ou, J.; Cheng, J.; Lin, C.; Ho, M. S.; Song, L.; He, Y.; Ou, J.; Zhao, Y.; Li, R.; Cheng, J.; Lin, C. Auxilin Underlies Progressive Locomotor Deficits and Dopaminergic Neuron Loss in a Drosophila Model of Parkinson ' s Disease Article Auxilin Underlies Progressive Locomotor Deficits and Dopaminergic Neuron Loss in a Drosophila Model of Parkinson ' s Disea. *CellReports* **2017**, *18*, 1132–1143.
20. Watanabe, S.; Mamer, L. E.; Raychaudhuri, S.; Luvsanjav, D.; Eisen, J.; Trimbuch, T.; Söhl-Kielczynski, B.; Fenske, P.; Milosevic, I.; Rosenmund, C.; Jorgensen, E. M. Synaptojanin and Endophilin Mediate Neck Formation during Ultrafast Endocytosis. *Neuron* **2018**, *98*, 1184-1197.e6.
21. Mani, M.; Lee, S. Y.; Lucast, L.; Cremona, O.; Di Paolo, G.; De Camilli, P.; Ryan, T. A. The Dual Phosphatase Activity of Synaptojanin1 Is Required for Both Efficient Synaptic Vesicle Endocytosis and Reavailability at Nerve Terminals. *Neuron* **2007**, *56*, 1004–1018.
22. Cremona, O.; Paolo, G. Di; Wenk, M. R.; Lu, A.; Kim, W. T.; Takei, K.; Daniell, L.; Nemoto, Y.; Shears, S. B.; Flavell, R. A.; Mccormick, D. A.; Camilli, P. De; Haven, N.; Mediche, S.; Carolina, N. Essential Role of Phosphoinositide Metabolism in Synaptic Vesicle Recycling. **1999**, *99*, 179–188.
23. Gong, L.; Camilli, P. De Regulation of postsynaptic AMPA responses by synaptojanin 1. **2008**, *105*.
24. Mcintire, L. B. J.; Berman, D. E.; Myaeng, J.; Staniszewski, A.; Arancio, O.; Paolo, G. Di; Kim, T. Reduction of Synaptojanin 1 Ameliorates Synaptic and Behavioral Impairments in a Mouse Model of Alzheimer ' s Disease. **2012**, *32*, 15271–15276.
25. Pan, X. P.; Li, X. X.; Wang, J.; Powell, X. J.; Wang, Q.; Zhang, X. Y.; Chen, Z.; Wicinski, B.; Hof, P.; Ryan, X. T. A.; Yue, Z. Parkinson ' s Disease-Associated LRRK2 Hyperactive Kinase Mutant Disrupts Synaptic Vesicle Trafficking in Ventral Midbrain Neurons. **2017**, *37*, 11366–11376.
26. Cossec, J.; Mircher, C.; Grattau, Y.; Hoischen, A.; Stora, S.; Olivomarin, J.; Chaumont, F. De; Lecourtois, M.; Antonarakis, S. E.; Veltman, J. A.; Delabar, J. M.; Duyckaerts, C.; Paolo, G. Di; Potier, M. Trisomy for Synaptojanin1 in Down syndrome is functionally linked to the enlargement of early endosomes. **2012**, *21*, 3156–3172.
27. Voronov, S. V.; Frere, S. G.; Giovedi, S.; Pollina, E. A.; Borel, C.; Zhang, H.; Schmidt, C.; Akeson, E. C.; Wenk, M. R.; Cimasoni, L.; Arancio, O.; Davisson, M. T.; Antonarakis, S. E.; Gardiner, K.; De Camilli, P.; Di Paolo, G. Synaptojanin 1-linked phosphoinositide dyshomeostasis and cognitive deficits in mouse models of Down's syndrome. *Proc. Natl. Acad. Sci. U. S. A.* **2008**, *105*, 9415–9420.
28. Cao, M.; Wu, Y.; Ashrafi, G.; McCartney, A. J.; Wheeler, H.; Bushong, E. A.; Boassa, D.; Ellisman, M. H.; Ryan, T. A.; De Camilli, P. Parkinson Sac Domain Mutation in Synaptojanin 1 Impairs Clathrin Uncoating at Synapses and Triggers Dystrophic Changes in Dopaminergic Axons. *Neuron* **2017**, *93*, 882-896.e5.
29. Zhou, W.; Barkow, J. C.; Freed, C. R. Running wheel exercise reduces α -synuclein aggregation and improves motor and cognitive function in a transgenic mouse model of Parkinson ' s disease. **2017**, 1–18.
30. Irrcher, I.; Aleyasin, H.; Seifert, E. L.; Hewitt, S. J.; Chhabra, S.; Phillips, M.; Lutz, A. K.; Rousseaux, M. W. C.; Bevilacqua, L.; Callaghan, S.; Maclaurin, J. G.; Winklhofer, K. F.; Rizzu, P.; Rippstein, P.; Kim, R. H.; Chen, C. X.; Fon, E. A.; Slack, R. S.; Harper, M. E.; Mcbride, H. M.; Mak, T. W.; Park, D. S. Loss of the Parkinson ' s disease-linked gene DJ-1 perturbs mitochondrial dynamics. **2010**, *19*, 3734–3746.
31. Aleyasin, H.; Rousseaux, M. W. C.; Phillips, M.; Kim, R. H.; Bland, R. J.; Callaghan, S.; Slack, R. S.; During, M. J.; Mak, T. W.; Park, D. S. The Parkinson ' s disease gene DJ-1 is also a key regulator of stroke-induced damage. **2007**.

32. Aleyasin, H.; Rousseaux, M. W. C.; Marcogliese, P. C.; Hewitt, S. J.; Irrcher, I.; Joselin, A. P. DJ-1 protects the nigrostriatal axis from the neurotoxin MPTP by modulation of the AKT pathway. **2009**.
33. Xu, S.; Yang, X.; Qian, Y.; Xiao, Q. Parkinson's disease-related DJ-1 modulates the expression of uncoupling protein 4 against oxidative stress. *J. Neurochem.* **2018**, *145*, 312–322.
34. Shi, H.; Deng, H.; Gius, D.; Schumacker, P. T.; Surmeier, D. J.; Ma, Y. Sirt3 protects dopaminergic neurons from mitochondrial oxidative stress. **2017**, *26*, 1915–1926.
35. Tai-Nagara, I.; Matsuoka, S.; Ariga, H.; Suda, T. Mortalin and DJ-1 coordinately regulate hematopoietic stem cell function through the control of oxidative stress. *Blood* **2014**, *123*, 41–50.
36. Lopert, P.; Patel, M. Redox Biology Brain mitochondria from DJ-1 knockout mice show increased respiration-dependent hydrogen peroxide consumption. *Elsevier* **2014**, *2*, 667–672.
37. Sheng, C.; Heng, X.; Zhang, G.; Xiong, R.; Li, H.; Zhang, S. DJ-1 deficiency perturbs microtubule dynamics and impairs striatal neurite outgrowth. **2013**, *34*, 489–498.
38. Giaime, E.; Yamaguchi, H.; Gautier, C. A.; Kitada, T.; Shen, J. Loss of DJ-1 Does Not Affect Mitochondrial Respiration but Increases ROS Production and Mitochondrial Permeability Transition Pore Opening. **2012**, *7*.
39. Shtifman, A.; Zhong, N.; Lopez, J. R.; Shen, J.; Xu, J. Altered Ca 2 + homeostasis in the skeletal muscle of DJ − 1 null mice. *Neurobiol. Aging* **2011**, *32*, 125–132.
40. Yamaguchi, H.; Shen, J. Absence of dopaminergic neuronal degeneration and oxidative damage in aged DJ-1-deficient mice. *Mol. Neurodegener.* **2007**, *2*, 10.
41. Burch, J.; McKenna, C.; Palmer, S.; Norman, G.; Glanville, J.; Sculpher, M.; Woolacott, N. Rimonabant for the treatment of overweight and obese people. *Health Technol. Assess.* **2009**, *13 Suppl 3*, 13–22.
42. Goldberg, M. S.; Pisani, A.; Haburcak, M.; Vortherms, T. A.; Kitada, T.; Costa, C.; Tong, Y.; Martella, G.; Tscherter, A.; Martins, A.; Bernardi, G.; Roth, B. L.; Pothos, E. N.; Calabresi, P.; Shen, J. Nigrostriatal dopaminergic deficits and hypokinesia caused by inactivation of the familial parkinsonism-linked gene DJ-1. *Neuron* **2005**, *45*, 489–496.
43. Pisani, A.; Martella, G.; Tscherter, A.; Costa, C.; Mercuri, N. B.; Bernardi, G.; Shen, J.; Calabresi, P. Enhanced sensitivity of DJ-1-deficient dopaminergic neurons to energy metabolism impairment : Role of Na + / K + ATPase. **2006**, *23*, 54–60.
44. Bonilha, V. L.; Bell, B. A.; Rayborn, M. E.; Yang, X.; Kaul, C.; Grossman, G. H.; Samuels, I. S.; Hollyfield, J. G.; Xie, C.; Cai, H.; Shadrach, K. G. Loss of DJ-1 elicits retinal abnormalities, visual dysfunction, and increased oxidative stress in mice. *Exp. Eye Res.* **2015**, *139*, 22–36.
45. Chandran, J. S.; Lin, X.; Zapata, A.; Höke, A.; Shimoji, M.; Moore, S. O.; Galloway, M. P.; Laird, F. M.; Wong, P. C.; Price, D. L.; Bailey, K. R.; Crawley, J. N.; Shippenberg, T.; Cai, H. Progressive behavioral deficits in DJ-1-deficient mice are associated with normal nigrostriatal function. *Neurobiol. Dis.* **2008**, *29*, 505–514.
46. Aron, L.; Klein, P.; Pham, T.; Kramer, E. R.; Wurst, W. Pro-Survival Role for Parkinson ′ s Associated Gene DJ-1 Revealed in Trophically Impaired Dopaminergic Neurons. **2010**, *8*.
47. Marcogliese, P. C.; Park, D. S.; Sanchez, G.; Varaschin, R. K.; Bu, H. Unaltered Striatal Dopamine Release Levels in Young Parkin Knockout , Pink1 Knockout , DJ-1 Knockout and LRRK2 R1441G Transgenic Mice. **2014**, *9*.
48. Oz, K.; Hiroki, T.; Yuri, M.; Ya, J. DJ-1 is indispensable for the S-nitrosylation of Parkin , which maintains function of mitochondria. **2020**, 1–11.
49. Cremer, J. N.; Amunts, K.; Schleicher, A.; Palomero-Gallagher, N.; Piel, M.; Rösch, F.; Zilles, K. Changes in the expression of neurotransmitter receptors in Parkin and DJ-1 knockout mice - A quantitative multireceptor study. *Neuroscience* **2015**, *311*, 539–551.
50. Hennis, M. R.; Seamans, K. W.; Marvin, M. A.; Casey, B. H.; Matthew, S. Behavioral and Neurotransmitter Abnormalities in Mice Deficient for Parkin , DJ-1 and Superoxide Dismutase. **2013**, *8*, 1–17.
51. Park, J.; Kim, S. Y.; Cha, G.; Lee, S. B.; Kim, S.; Chung, J. Drosophila DJ-1 mutants show

oxidative stress-sensitive locomotive dysfunction. **2005**, *361*, 133–139.
52. Vingill, S.; Brockelt, D.; Lancelin, C.; Tatenhorst, L.; Dontcheva, G.; Preisinger, C.; Schwedhelm-domeyer, N.; Joseph, S.; Mitkovski, M.; Nave, K.; Schulz, J. B.; Marquardt, T.; Lingor, P.; Stegmüller, J. Loss of FBXO 7 (PARK 15) results in reduced proteasome activity and models a parkinsonism-like phenotype in mice. **2016**, *35*, 2008–2025.
